# Multiple intersecting pathways are involved in CPEB1 phosphorylation and regulation of translation during mouse oocyte meiosis

**DOI:** 10.1242/dev.202712

**Published:** 2024-06-04

**Authors:** Chisato Kunitomi, Mayra Romero, Enrico Maria Daldello, Karen Schindler, Marco Conti

**Affiliations:** ^1^ Center for Reproductive Sciences, University of California, San Francisco, CA 94143, USA; ^2^ Eli and Edythe Broad Center of Regeneration Medicine and Stem Cell Research, University of California, San Francisco, CA 94143, USA; ^3^ Department of Obstetrics and Gynecology and Reproductive Sciences, University of California, San Francisco, CA 94143, USA; ^4^ Rutgers, The State University of New Jersey, Piscataway, NJ 08854, USA; ^5^ Human Genetics Institute of New Jersey, Piscataway, NJ 08854, USA; ^6^ Sorbonne Université, CNRS, Laboratoire de Biologie du Développement - Institut de Biologie Paris Seine, LBD - IBPS, F-75005 Paris, France

**Keywords:** CPEB1, mRNA, Meiosis, Oocyte, Translation

## Abstract

The RNA-binding protein cytoplasmic polyadenylation element binding 1 (CPEB1) plays a fundamental role in regulating mRNA translation in oocytes. However, the specifics of how and which protein kinase cascades modulate CPEB1 activity are still controversial. Using genetic and pharmacological tools, and detailed time courses, we have re-evaluated the relationship between CPEB1 phosphorylation and translation activation during mouse oocyte maturation. We show that both the CDK1/MAPK and AURKA/PLK1 pathways converge on CPEB1 phosphorylation during prometaphase of meiosis I. Only inactivation of the CDK1/MAPK pathway disrupts translation, whereas inactivation of either pathway alone leads to CPEB1 stabilization. However, CPEB1 stabilization induced by inactivation of the AURKA/PLK1 pathway does not affect translation, indicating that destabilization and/or degradation is not linked to translational activation. The accumulation of endogenous CCNB1 protein closely recapitulates the translation data that use an exogenous template. These findings support the overarching hypothesis that the activation of translation during prometaphase in mouse oocytes relies on a CDK1/MAPK-dependent CPEB1 phosphorylation, and that translational activation precedes CPEB1 destabilization.

## Teaser

**Summary:** The activation of mRNA translation up to metaphase I in mouse oocytes requires CDK1 phosphorylation but not CPEB1 destabilization.

## INTRODUCTION

Completion of meiosis and generation of female haploid gametes competent for fertilization are developmental processes that are essential for propagation of the species. In mammals, female meiosis initiates during fetal development, when chromosomes replicate and homologous recombination is initiated (Seydoux and Braun, 2006). After oocytes have undergone chromosome pairing and homologous recombination, they arrest meiotic progression and enter a prolonged period of cell cycle quiescence (Gosden and Lee, 2010). This period of quiescence ends when fully grown oocytes are enclosed in antral follicles and are ready to be ovulated. Meiotic cell-cycle resumption is triggered by the luteinizing hormone (LH) surge followed by the relief of a somatic paracrine repressive signal that maintains meiotic arrest (Mehlmann, 2005; Conti et al., 2012; Conti and Franciosi, 2018). During the growth and arrest period, oocytes store mRNAs that, upon meiotic, resumption are polyadenylated and translated. Protein kinases that regulate meiotic maturation also play a role in controling translation. How protein kinase signaling, the cell cycle and translation are coordinated in mammalian oocytes remains unclear.

Cyclin-dependent kinase 1 (CDK1) bound to cyclin B is the driver of meiotic re-entry (Kishimoto, 2018). During quiescence, CDK1 activity is inhibited by WEE2-dependent phosphorylation (Han et al., 2005). Upon the LH surge-mediated decline in cAMP, CDC25 phosphatase becomes activated and translocates into the nucleus, thereby activating CDK1 (Pirino et al., 2009; Oh et al., 2010). Active nuclear CDK1 triggers nuclear envelope breakdown (GVBD) and chromosome condensation (Ubersax et al., 2003). These processes, and spindle assembly, are all steps that are necessary for the prophase-to-metaphase I (MI) transition (Holt et al., 2013). Another signaling pathway that controls meiotic maturation is the MOS/ERK pathway. Although activation of MOS is required for meiotic resumption in *Xenopus* oocytes (Gotoh et al., 1995; Ferrell, 1999), meiotic re-entry occurs in *Mos*-null mice (Colledge et al., 1994) and when the downstream ERK1/ERK2 kinases are genetically inactivated (Sha et al., 2017). Positive feedback between MAPK and CDK1 has been described and is involved at different times during *Xenopus* oocyte meiotic progression (Abrieu et al., 2001). These interactions are essential for meiotic spindle properties, for the asymmetry of the first meiotic division and for maintaining metaphase II (MII) arrest (Kishimoto, 2003; Jessus et al., 2020). In contrast to *Xenopus*, the role of MOS/MAPK during early stages of meiosis I in mouse is less clear. However, several defects in spindle organization and localization are associated with MAPK loss of function (Verlhac et al., 2000) (Sha et al., 2017). Independent of CDK1 and MOS, an Aurora kinase signaling pathway functions in early in maturation. Aurora kinase A (AURKA) is activated around microtubule-organizing centers (MTOCs) (Blengini et al., 2021). AURKA activation then phosphorylates PLK1 (Baran et al., 2016; Solc et al., 2015; Joukov and De Nicolo, 2018). Small molecular inhibitors and genetic manipulation of AURKA document that this pathway is essential for spindle function and meiotic progression (Blengini et al., 2022).

Although these cascades of phosphorylation play pivotal roles in meiotic re-entry, regulation of mRNA translation and synthesis of components that are crucial for meiotic progression are also indispensable for meiotic re-entry in *Xenopus* and for meiotic progression in mouse. For example, in *Xenopus* oocytes, translation of *Mos* mRNA is followed by activation of the MAPK pathway, thus signaling oocyte meiotic re-entry. This translation may be triggered by cyclin-independent activation of CDK1 (Kim and Richter, 2007). In addition to translational regulation of *Mos*, cyclin synthesis is also crucial for sustaining CDK1 activity (Mendez and Richter, 2001; Conti and Kunitomi, 2024). RNA-binding proteins such as MUSASHI and CPEB1 play crucial functions in translational regulation throughout oocyte maturation both *Xenopus* and mouse (Charlesworth et al., 2006; Mendez and Richter, 2001). In *Xenopus* and mouse oocytes, CPEB1 phosphorylation is required to assemble a complex that promotes polyadenylation of mRNA and its translational activation (Richter and Lasko, 2011). Numerous studies have been published on the phosphorylation of CPEB1 and the consequent translational regulation in *Xenopus*. It was initially demonstrated that Aurora A/Eg2 kinase phosphorylates CPEB1 (Mendez et al., 2000b; Mendez et al., 2000a). However, additional studies have questioned a role for this kinase in CPEB1 phosphorylation (Keady et al., 2007; Radford et al., 2008; Frank-Vaillant et al., 2000). Later during MI, CPEB1 receives additional phosphorylation by CDK1/ERK that signal its destabilization (Mendez et al., 2002). This destabilization is proposed to cause an activation of translation of mRNAs such as *Ccnb1* but not *Mos*. In addition to CDK1, PLK1-mediated phosphorylation may also trigger CPEB1 degradation (Setoyama et al., 2007). The kinases involved in CPEB1 phosphorylation in mammalian oocytes are equally unclear. Initial studies indicated that AURKA also phosphorylated CPEB1 in mouse oocytes (Hodgman et al., 2001). However, follow-up studies using pharmacological inhibition concluded that AURKA does not phosphorylate CPEB1 or control translational activation in pig and mouse oocytes (Komrskova et al., 2014; Han et al., 2017). More-recent genetic studies using a null *Aurka* allele have re-proposed a role for this kinase in translational regulation in mouse oocytes (Aboelenain and Schindler, 2021). In addition, as in *Xenopus*, CDK1-dependent phosphorylation of CPEB1 in mouse oocytes is likely responsible for its degradation around MI (Tay et al., 2000). Others have proposed that MAP kinase-dependent phosphorylation is the most relevant pathway that signals CPEB1 degradation (Sha et al., 2017) and activation of translation (Sha et al., 2017; Zhang et al., 2018). Here, we have re-assessed the different signaling pathways converging on CPEB1 phosphorylation and translational activation in mouse oocytes. We also integrate the timing of these molecular events with cellular events that occur during meiosis, such as GVBD. Our findings show that translation occurs before AURKA-dependent CPEB1 destabilization, and that CDK1/MAPK are essential for mRNA translation.

## RESULTS

### Translational activation of CcnB1 and Mos mRNAs requires CPEB1

**Fig. 1.**
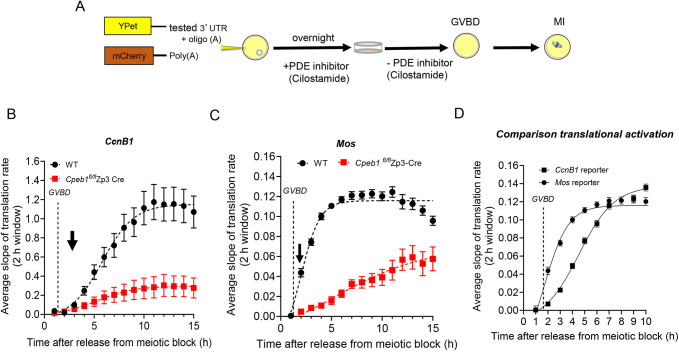
**CPEB1 is required for the translational activation of *Ccnb1* and *Mos* mRNAs.** (A) Schematic representation of the YFP reporter assay, using the Ypet variant. GV-arrested oocytes were injected with 5-10 pl of 12.5 ng/µl cyclin B1 (*Ccnb1*) or *Mos* reporter and 12.5 ng/µl polyadenylated mCherry. After overnight incubation with 1 µM cilostamide (a PDE inhibitor), oocytes were released from the meiotic block. YFP and mCherry signals were recorded during maturation by time-lapse microscopy every 15 min for 15 h. The translation rate for each oocyte was calculated by linear regression of the reporter data using a 2 h moving window and the mean±s.e.m. of all the oocytes from three independent experiments was plotted against incubation time. (B) Translation rates of the *Ccnb1* reporter in wild-type and *Cpeb1*^fl/fl^Zp3-cre oocytes during maturation (difference between the two groups: *P*<0.001 by nonparametric Mann–Whitney test). (C) Translation rates of the *Mos* reporter in wild-type and *Cpeb1*^fl/fl^Zp3-cre oocytes during maturation (difference between the two groups: *P*<0.00001 by nonparametric Mann–Whitney test) (D) Comparison of the timing of *Ccnb1* and *Mos* translation rates in wild-type oocytes during maturation. The data are mean±s.e.m. Nuclear envelope breakdown (GVBD) timing (average 1.75 h) is shown in the graph. The arrows in B and C indicate the first point in the time course when a change in the rate of translation is significant. Results are significantly different at *P*<0.00001 (nonparametric Mann–Whitney test).

Given the pivotal roles of cyclin B1 (CCNB1) and MOS in regulating meiotic cell-cycle progression, several reports have investigated the transcriptional and post-transcriptional regulatory mechanisms governing the expression of these proteins. Previous studies explored the involvement of the RNA-binding protein CPEB1 in the translational activation of cyclin genes and *Mos* mRNAs in mouse oocytes, using *in vitro* methods of *Cpeb1* siRNA knockdown or mutagenesis of the 3′ untranslated region (UTR) of target mRNAs (Tay et al., 2000; Yang et al., 2017; Han et al., 2017). Here, we have employed *in vivo* genetic manipulations to assess how CPEB1 is involved in the translational activation of *Ccnb1* and *Mos* mRNAs. Cyclin and *Mos* reporter mRNAs, in which the 3′ UTR control the accumulation of the fluorescent protein YFP, were co-injected with the *mCherry* reporter into prophase I-arrested (GV) mouse oocytes isolated from wild-type and CPEB1 oocyte-specific knockout (*Cpeb1*^fl/fl^ Zp3-cre) mice (Fig. 1A). We then monitored reporter accumulation during oocyte maturation using time-lapse microscopy (Fig. S1A-D). When assessing changes in *Ccnb1* and *Mos* translation, we detected an increase in rates during oocyte maturation in oocytes from wild-type controls (Fig. 1B,C); however, translation rates for both were significantly decreased in oocytes derived from *Cpeb1^fl/fl^* Zp3-cre mice (Fig. 1B,C) (FDR<0.0001). As a control, we found that translation of a reporter that uses the short *Ccnb1* 3′UTR, which is devoid of CPEB1-binding sites (Yang et al., 2017), was not affected in *Cpeb1^fl/fl^* Zp3-cre oocytes (Fig. S1E). Upon examining the timing of reporter accumulation in more detail, an increase in translation rate became significant 2-3 h after release from cilostamide (PDE inhibitor) block (30 min-1 h after GVBD) in oocytes from wild-type mice (Fig. 1B,C, downward arrow). This timing is similar or slightly delayed compared with ribosome loading onto endogenous mRNAs encoding the two proteins (Luong et al., 2020). Ribosome loading increased immediately after GVBD at the 2 h time point (Han et al., 2017; Luong et al., 2020) (Fig. S2). Of note, the timing of translational activation of these mRNAs after GVBD differs from that observed in *Xenopus* oocytes, where *Mos* mRNA translation is activated before GVBD, and *Ccnb1* mRNA translation is delayed until prometaphase (Mendez et al., 2002; Pique et al., 2008). Despite activation of both occurring post-GVBD in mice, a difference in the timing of translational activation of the two reporters was detected. We found that translational activation of *Mos* mRNA preceded that of *Ccnb1* (Fig. 1D), a difference reminiscent of that described in *Xenopus* oocytes. Moreover, although some CPEB1 protein remains in oocytes from *Cpeb1^fl/fl^* Zp3-cre mice (Fig. S1F) (Luong et al., 2020), our data still support the conclusion that CPEB1 plays a major role in the translation of both *Ccnb1* and *Mos* mRNAs.

### Multiple pathways control phosphorylation of CPEB1 in oocytes

To delve further into the different molecular events necessary for translational activation during oocyte meiotic re-entry, we reconsidered the different phosphorylation cascades reported to converge on CPEB1 and translational regulation. To probe the time dimension of these events, we used both loss of function and small-molecule pharmacological inhibition approaches to investigate the contribution of pathways leading to CPEB1 phosphorylation and translational activation. Although in previous reports we used a pharmacological strategy to manipulate relevant kinases (Han et al., 2017; Luong et al., 2020), here we introduced two major modifications in the experimental protocol. First, we used culture conditions in which an oil overlay was omitted during oocyte incubation, because the hydrophobicity of the small-molecule inhibitors used may partition to the oil phase (Rémillard Labrosse et al., 2024). Second, because inhibition of any of these pathways prevents oocyte progression to metaphase II (MII), all measurements were performed at 7-8 h of incubation, when the oocytes are still in metaphase I (MI). This latter change in experimental protocol should remove the confounding variable of comparing oocytes at different stages of the meiotic cell cycle depending on the treatment. Using these experimental settings, we focused on two major interconnected pathways converging on CPEB1: the AURKA/PLK1 and CDK1/MOS-ERK pathways.

To determine whether these pathways directly or indirectly converge onto the phosphorylation status of CPEB1, we assessed the changes in CPEB1 electrophoretic mobility using SDS-PAGE. A phosphoproteome analysis (Cheng et al., 2022) detected multiple CPEB1 phosphorylation sites (phospho-sites) in mouse oocytes (Fig. S3A). These phospho-sites fall within CDK1, MAPK, AURKA and PLK1 consensus sites. We and others have shown that multiple stepwise changes in mobility and generation of different CPEB1 immunoreactive forms are detected during mouse oocyte maturation (Tay et al., 2000; Han et al., 2017; Yang et al., 2017). A minor shift in CPEB1 immunoreactivity during the first hour of oocyte culture was observed only under some electrophoretic conditions (Han et al., 2017), but a more significant mobility shift was consistently observed at 2 h. We refer to this CPEB1 species as the phosphorylated form (Fig. S3B). This shift is followed by additional decreases in mobility between 3 and 5 h (Fig. S3B). As in other reports (Mendez et al., 2002), we refer to the slowest migrating form at 4 h as the hyper-phosphorylated form.

**Fig. 2.**
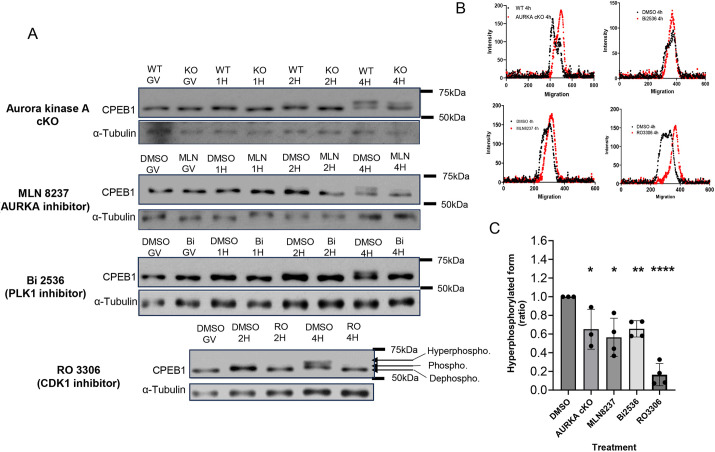
**CPEB1 is phosphorylated through activation of multiple pathways.** (A) Representative western blot images of the time course of CPEB1 phosphorylation with different treatments. Western blot analyses were conducted on lysates of 30 oocytes at the reported time points after release from the meiotic block. α-Tubulin or DDB1 was used as a loading control. (B) The profile of band intensity at 4 h in control (black dots) and experimental (red squares) groups are plotted against the migration on the gel. (C) The integration of hyper-phosphorylated peak intensities was calculated and compared with DMSO. Each point is the ratio of experimental/DMSO control and represents a different experiment and a different biological replicate of the three or more experiments performed. A two-tailed unpaired Student's test was used to evaluate the statistical significance between DMSO and each treatment (*P<0.05, **P<0.01, ****P<0.0001). With the exception of the RO3306 profile, the abundance of the hyper-phosphorylated band in each treatment is overestimated because it is not completely resolved from the phosphorylated band. WT, wild type; KO, knockout; GV, prophase I-arrested oocytes; AURKA, Aurora kinase A.

When using this mobility readout to assess the effect of inhibition of the different signaling pathways, we found that the mobility of the CPEB1 immunoreactive band was clearly affected in oocytes derived from oocyte-specific *Aurka* KO (conditional; cKO) mice (Fig. 2A). Specifically, and although the generation of the phosphorylated form was not affected (Fig. S4), the accumulation of the hyper-phosphorylated form was delayed and the ratio of phospho/hyper-phosphorylated forms at 4 h of meiotic maturation was inverted: wild-type oocytes accumulated more of the hyper-phosphorylated form, whereas more of the phosphorylated form was present in *Aurka* cKO oocytes (Fig. 2B,C). The involvement of AURKA in generating this phosphorylation pattern was confirmed by inhibition of AURKA activity with a concentration of MLN8237 that preferentially block this kinase (Blengini et al., 2022) (Fig. 2A-C). This small-molecule inhibitor mimicked the genetic loss of function, producing less of the hyper-phosphorylated form at 4 h (Fig. 2A-C). Pharmacological inhibition of PLK1 by Bi2536, a kinase phosphorylated and activated by AURKA (Willems et al., 2018), produced an even more profound effect, and the hyper-phosphorylated form was not detected at 4 h in some experiments (Fig. 2A-C). Control experiments where AURKA activation via T-loop phosphorylation was monitored by western blotting confirmed a complete inhibition by these treatments (Fig. S5B). Thus, and unlike previous conclusions (Komrskova et al., 2014; Han et al., 2017), AURKA/PLK1 either directly or indirectly affects the phosphorylation state of CPEB1.

Several CPEB1 phospho-peptides containing CDK1 consensus sites have previously been identified by mass spectrometry (Fig. S3A). Inhibition of CDK1 activity with the specific inhibitor RO3306 prevented the generation of both the phosphorylated and hyper-phosphorylated forms (Fig. 2A-C). Because CDK1 and MOS pathway activation are intertwined (Abrieu et al., 2001; Cao et al., 2020), we performed western blotting to monitor ERK phosphorylation and found that inhibition of CDK1 prevented ERK activation (Fig. S5C).

Because AURKA/PLK1 and CDK1/MAPK pathways intersect at several steps through positive and negative feedback, we tested whether inhibition of AURKA signaling affects CDK1 activity by assessing GVBD timing and MAPK activation by measuring ERK phosphorylation status. When assessing GVBD timing by time lapse microscopy, we observed a 30 min delay in oocyte GVBD in *Aurka* cKO oocytes and upon AURKA pharmacological inhibition (Fig. S5A), suggesting that altered phosphorylation around MTOCs delays CDK1 activation. However, MAPK phosphorylation was not decreased in *Aurka* cKO oocytes, or by MLN8237 and Bi2536 treatments, which inhibit AURKA and PLK1, respectively (Fig. 4C, Fig. S5C). Thus, it is unlikely that the altered CPEB1 phosphorylation that follows manipulation of AURKA signaling is due to altered CDK1 function. CDK1 inhibition completely blocked MAPK phosphorylation, a finding consistent with the view that CDK1 activation functions upstream of MAPK activation, likely through control of *Mos* mRNA translation (Fig. S5C) or MOS protein stabilization (Castro et al., 2001; Santoni et al., 2024). In summary, these experiments indicate that timely phosphorylation of CPEB1 during prometaphase is dependent on both functional AURKA and CDK1 pathways.

### Inhibition of phosphorylation of CPEB1 causes stabilization of the protein and/or delayed degradation

**Fig. 3.**
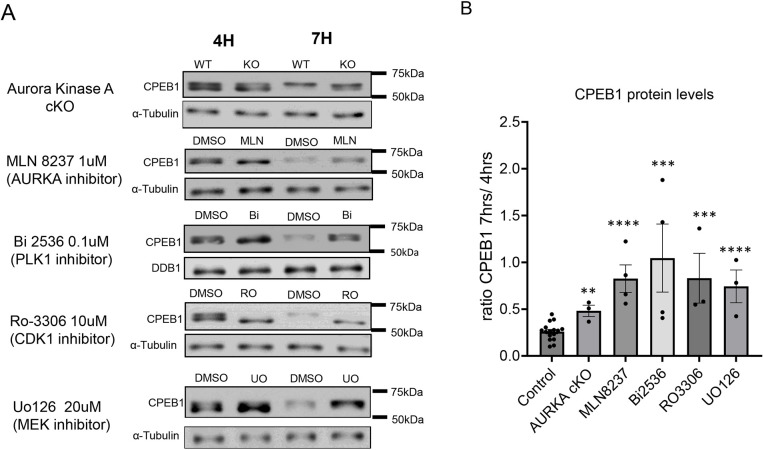
**CPEB1 stabilization is induced by the inhibition of different protein kinases.** (A) Representative western blot images of the CPEB1 phosphorylation 4 and 7 h after release from the meiotic block in the AurkA cKO oocytes or after different treatments. Western blot analysis was conducted on lysates of 30 oocytes. α-Tubulin or DDB1 was used as a loading control. The blots shown here were re-probed with either phospho-AurkA antibodies for the images in Fig. S5B or with phospho-Erk antibodies for the MLN8237, Bi2536 and RO-3306 images in Fig.S5C. (B) CPEB1 stabilization was calculated as the ratio of the CPEB1 immunoreactive signal between 7 h and 4 h from the meiotic block release. Data are mean±s.e.m. of three or more independent biological replicates. Two-tailed unpaired Student's test was used to evaluate the statistical significance between DMSO and each treatment (**P<0.01, ***P<0.001, ****P<0.0001). WT, wild type; KO, knockout; AURKA, Aurora kinase A.

Previous reports indicated that hyper-phosphorylation of CPEB1 is associated with its destabilization (Mendez et al., 2002). This post-translational modification is proposed as a mechanism of translational activation because CPEB1 represses 3′UTRs that contain multiple CPEs (Mendez et al., 2002; Sha et al., 2017). We then determined whether the manipulation of the above pathways also affects the stability of CPEB1 in MI (Fig. 3A,B). Both genetic and pharmacological inhibition of the AURKA/PLK1 pathway caused partial or complete stabilization of CPEB1 protein at 7 h (Fig. 3A,B). Although hyper-phosphorylation was still present in *Aurka* cKO oocytes and after treatment of wild-type oocytes with MLN8237 at 7 h, its levels were decreased in PLK1 inhibited oocytes (Fig. 3A). As mentioned previously, the loss of AURKA function caused a 30-40 min delay in GVBD, and similar effects were detected with MLN8237 and Bi2536 treatments (Fig. S5A). This short delay in cell-cycle re-entry did not impact the time course of degradation because CPEB1 stabilization after PLK1 inhibition was detected at 7-8 h, a time when oocytes reached MI (Fig. S6). Loss of both phosphorylated and hyper-phosphorylated forms, and CPEB1 stabilization in the unphosphorylated state were evident with CDK1 inhibition (Fig. 3A). Stabilization was also observed with the MAPK inhibitors; however, the migration of the stabilized form corresponded to that of a hyper-phosphorylated CPEB1 (Fig. 3A,B). Taken together, these findings are consistent with the hypothesis that activation of both pathways is involved in CPEB1 hyper-phosphorylation and destabilization.

### Inhibition of the AURKA pathway does not compromise Ccnb1 mRNA translation and CCNB1 protein accumulation

**Fig. 4.**
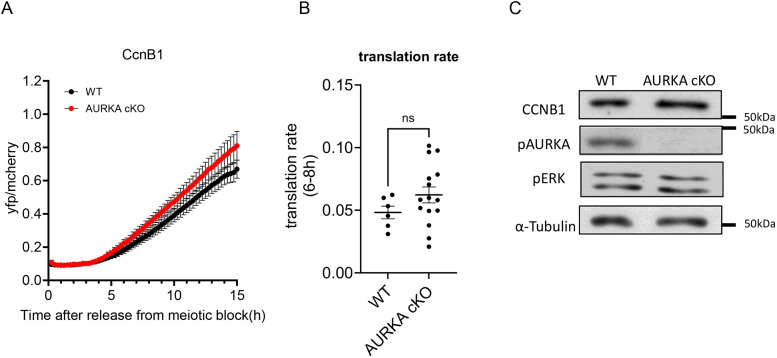
**Loss of function of Aurora kinase A does not compromise *Ccnb1* mRNA translation and endogenous CCNB1 protein accumulation.** GV-arrested oocytes were injected with cyclin B1 (Ccnb1) reporter and polyadenylated mCherry mRNAs. After overnight incubation, oocytes were released in medium free of PDE inhibitor (cilostamide). YFP and mCherry signals were recorded during maturation by time-lapse microscopy every 15 min for 15 h. (A) The YFP/mCherry signal ratio for each oocyte was plotted and data are shown as the mean±s.e.m. (B) Translation rates were calculated for each oocyte by linear regression of the reporter data between 6 and 8 h after incubation. A two-tailed unpaired Student's test was used to evaluate statistical significance. ns, not significant. (C) Western blotting was carried out on lysates of 50 oocytes from wild-type and Aurora kinase A (AURKA) cKO mice after 7 h of maturation. α-Tubulin was used as a loading control. The intensity of the endogenous CCNB1 and pERK bands was not significantly different between wild type and Aurka cKO.

**Fig. 5.**
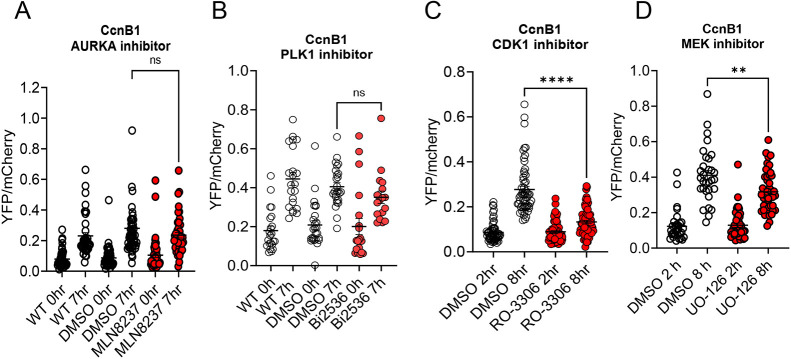
**Effect of different inhibitors on *Ccnb1* translation.** GV-arrested oocytes were injected with a cyclin B1 (*Ccnb1*) reporter and polyadenylated *mCherry*, and allowed to recover overnight. (A,B) For MLN8237 and Bi2536 experiments, microinjected oocytes were pre-incubated for 1 h with inhibitors (final concentrations: MLN8237, 1 µM; Bi2536, 0.1 µM) before releasing the oocytes into PDE inhibitor (cilostamide)-free medium containing the drugs. (C,D) For the RO-3306 (10 µM) and UO-126 (20 µM) experiments, oocytes were injected and released into cilostamide-free medium to undergo nuclear envelope breakdown (GVBD). Oocytes that underwent GVBD were collected and transferred to a medium including cilostamide. In all experiments, YFP and mCherry signals were plotted at 0 or 2 h incubation and 7-8 h. The data are mean±s.e.m. from three different experiments, with each dot representing one oocyte. A two-tailed unpaired Student's test was used to evaluate statistical significance (ns, not significant; **P<0.01, ****P<0.0001). AURKA, Aurora kinase A; WT, wild type.

Given the prominent function of CPEB1 in translational activation during oocyte maturation, we next investigated how interference with any of the above phosphorylation pathways would affect translation of *Ccnb1* mRNA, which is a prototypic target of this RNA-binding protein (RBP). Genetic ablation of *Aurka* had no significant effect on the translational activation of *Ccnb1* reporter translation (Fig. 4A) and the rate of translation in the absence of AURKA activity at 6-8 h was not significantly different from wild-type controls (Fig. 4B). The absence of any effect when AURKA was not present was confirmed even when protracting the incubation for 15 h (Fig. 4A). Similarly, activation of translation in the presence of AURKA or PLK1 inhibitors (MLN8237 or Bi2536, respectively) was not significantly reduced (Fig. 5A,B), even though a trend toward a decrease was detected with PLK1 inhibition. Conversely, CDK1 inhibition caused a profound decrease in translational activation when applied immediately after GVBD (Fig. 5C). The delayed treatment was necessary to allow oocytes to undergo GVBD. An 80% decrease in translation rate was observed at 8 h (Fig. 5C). The translation rate was further assessed in a complete time course up to 15 h (Fig. S7A,B). Some late activation was observed but it could not be determined whether this is due to inactivation of the drug during time-lapse microscopy or to activation of alternative translational activation pathways. MAPK inhibitors had some effects (Fig. 5D) but these were not as profound as those induced by CDK1 inhibition (Fig. S7E-H). At present, we cannot determine whether the effects of CDK1 on translation are direct or entirely mediated by activation of MAPK.

**Fig. 6.**
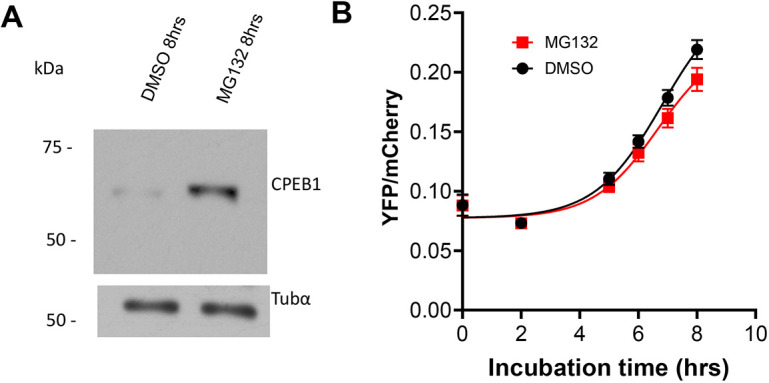
**Stabilization of CPEB1 through inhibition of the proteasome does not affect *Ccnb1* translation.** Oocytes were harvested and injected with the CcnB1-3′UTR reporter and mCherry, as described in the Materials and Methods. After overnight recovery, fluorescence intensity was recorded in quiescent oocytes (GV). Oocytes were then washed free of inhibitors and allowed to progress through maturation for 2 h. Oocytes that had undergone GVBD were selected, and the proteasome inhibitor Mg132 (10 μM) was added to half of the oocytes, while the other half received vehicle (DMSO). Time-lapse microscopy was started and the fluorescence intensity in each oocyte was recorded for a further 6 h. (A) At the end of incubation, a group of oocytes was saved for western blot using Cpeb1 or α-tubulin antibodies. (B) The YFP/mCherry ratio (mean±s.e.m.) of three separate experiments carried out with different sets of oocytes (total number of oocytes used: DMSO=62, Mg132=63) is reported. Only the 8 h time point reached statistical significance when using a Mann–Whitney test (q value=0.0479).

Because the above data indicate that degradation of CPEB1 and translational activation can be dissociated, we used a different experimental paradigm to further test this possibility. CPEB1 is degraded through ubiquitylation and subsequent proteosome degradation. Therefore, we used the MG132 proteasome inhibitor to block CPEB1 degradation. This treatment prevented degradation of CPEB1 at MI (Fig. 6). Again, CPEB1 stabilization did not have a significant effect on translation activation of the *Ccnb1* reporter (Fig. 6).

**Fig. 7.**
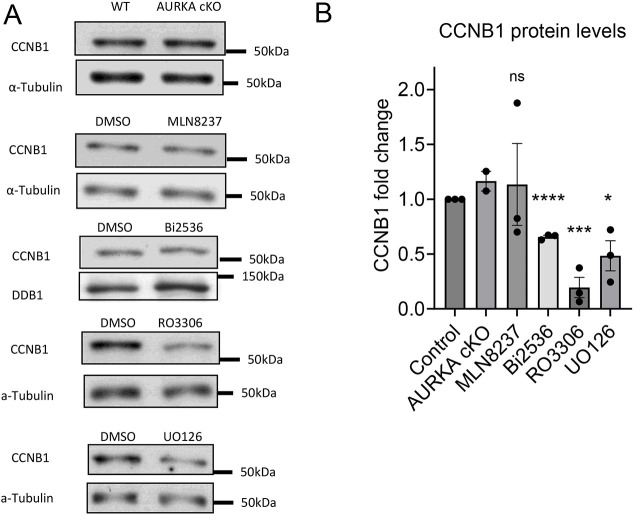
**Endogenous CCNB1 protein accumulation with different inhibitors.** (A) Cyclin B1 protein (CCNB1) accumulation in MI oocytes (7 h incubation) with different treatments. α-Tubulin or DDB1 was used as a loading control. 50-75 oocytes per lane were loaded for each experiment. (B) Quantification of western blot run in the above conditions. The data are CCNB1/α-tubulin or DDB1 ratios, and are expressed as fold changes over DMSO control. Data are mean±s.e.m. of three independent biological. A two-tailed unpaired Student's test was used to evaluate the statistical significance between the control and each treatment (ns, not significant; *P<0.05, ***P<0.001, ****P<0.0001). AURKA, Aurora kinase A; WT, wild type.

To confirm the divergent effect of the two kinase pathways on translation of the reporter, we measured the levels of endogenous CCNB1 accumulation (Fig. 7). CCNB1 protein levels were consistent with the reporter assay, whereby CDK1 inhibition had the most profound effects, followed by MAPK inhibitors: AURKA or PLK1 inhibition had no or only minor effects on CCNB1 accumulation in MI (Figs 4C, 7). Therefore, translation of endogenous mRNA and of a reporter consistently show that the AURKA/PLK1 pathway does not affect CCNB1 translation, regardless of stabilizing CPEB1.

### Mos mRNA translation is not affected by suppression of the AURKA/PLK1 pathway

**Fig. 8.**
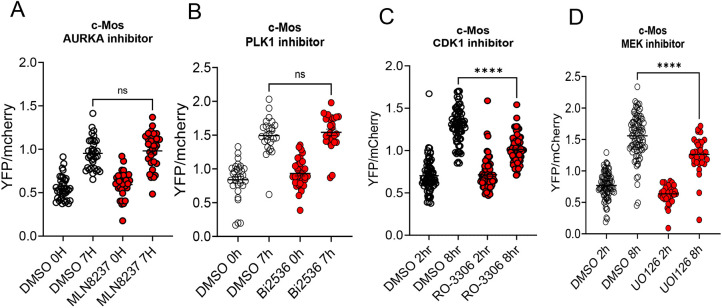
**Translation of *Mos* reporter in oocytes treated with different inhibitors.** GV-arrested oocytes were injected with *Mos* reporter and polyadenylated *mCherry* mRNAs. (A,B) For the experiments with MLN8237 (1 µM) or Bi2536 (0.1 µM), microinjected oocytes were pre-incubated for 1 h with inhibitors before releasing the oocytes to PDE inhibitor (cilostamide)-free medium containing the inhibitor. (C,D) For the RO-3306 (10 µM) and UO-126 (20 µM) experiments, oocytes were injected and released to a cilostamide-free medium to undergo nuclear envelope breakdown (GVBD). Oocytes that underwent GVBD were collected and transferred to the inhibitor-containing medium. In all experiments, YFP and mCherry signals were plotted at 0 h or 2 h incubation (the time when the oocytes were transferred to cilostamide-free drug-containing medium) and 7-8 h of incubation. The data are plotted as the mean±s.e.m. of the rate of reporter accumulation. A two-tailed unpaired Student's test was used to evaluate statistical significance (ns, not significant; ****P<0.0001). AURKA, Aurora kinase A.

To verify whether the divergent effect of the two kinase pathways is exclusive to cyclin B1 mRNA translation, we repeated the translation experiments with a *Mos* reporter (Fig. 8). Again, we found that only CDK1 and/or MAPK inhibition had a significant effect on *Mos* reporter translation. Further analysis of *Mos* translation after CDK1 inhibition indicated that no activation occurred (Fig. S7C,D), with accumulation of the reporter throughout maturation occurring at a constant rate. This finding indicates that *Mos* mRNA translational activation is entirely CDK1 dependent.

### Time and coordination of different events leading to oocyte progression to MI

**Fig. 9.**
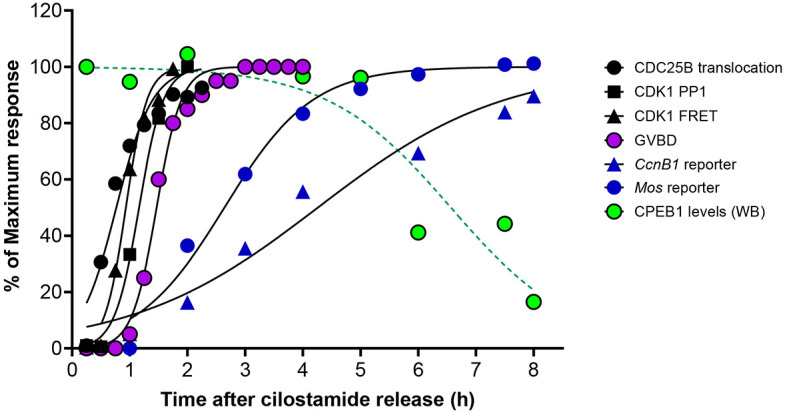
**Summary of the timing of landmark events of mouse oocyte *in vitro* maturation to metaphase I.** The CDC25B protein translocation into the nucleus, which is a proxy of inactivation of the CAMP/PKA pathway, was followed in oocytes microinjected with fluorescence-tagged CDC25B. The CDK1 activity was measured in live oocytes using a FRET reporter or by measuring CDK1 activity in oocyte lysates by monitoring the phosphorylation of a PP1 peptide substrate. The timing of CDK1 activation using the two measurements is comparable. Nuclear envelope breakdown (GVBD) was monitored by time-lapse microscopy and is the average of measurements of more than 100 oocytes. The change in rate accumulation of the *Mos* and *Ccnb1* reporter during oocyte maturation was followed by time-lapse microscopy after microinjection of mRNAs encoding the reporters fused to the corresponding 3′UTR. CPEB1 protein levels were assessed in extracts from oocytes cultured for different times after removal of the meiotic block. Each point is the mean of four independent experiments. Raw data for each parameter measured is reported in Figs S8 and S9. All data are normalized to the maximum value and reported as a percentage of the maximum. In all cases, the s.e.m. is omitted to improve clarity. Lines are the best fit of the experimental data using a four-parameters logistic equation and variable slope. Average time to reach 50% of the maximum in hours (±95 confidence limit): CDC25B=0.7674 (0.6199 to 0.9234); CDK1 activity measured by PP1 *in vitro* phosphorylation=1.161 (1.092 to 1.232); CDK1 activity measured by FRET in live oocytes=0.9326 (0.8460 to 1.023); GVBD= 1.466 (1.417 to 1.517); *Mos* reporter accumulation=2.640 (2.360 to 2.928); *Ccnb1* reporter accumulation=4.260 (3.660 to 4.898); CPEB1 levels=6.566 (5.701 to 7.562). The rate of translation of the reporters is comparable with the timing of increased ribosome loading (see Fig. S2).

Our data show that CDK1 is essential for phosphorylation of CPEB1 and for translation during meiotic maturation. To integrate the timing of these events, we compared their timing with oocyte meiotic re-entry and progression to MI (Fig. 9). These events included CDC25 translocation into the GV, CDK1 activation, GVBD, translational activation of *Mos* and *Ccnb1*, and a decline in CPEB1 levels. This comparison underscores other observations that activation of translation of these mRNAs precedes CPEB1 degradation.

## DISCUSSION

Here, we present experimental evidence of a convergence of the AURKA/PLK1 and CDK1/MAPK signaling pathways on phosphorylation of the RNA-binding protein CPEB1. However, in mouse oocytes, only the CDK1/MAPK pathway is essential for translational activation of an endogenous mRNA and of a microinjected reporter during the transition from prophase to metaphase I (MI). Consistent with previous findings, we show that hyper-phosphorylation of CPEB1 leads to its destabilization and degradation. Under conditions defined in this study, preventing CPEB1 destabilization induced by AURKA/PLK1 phosphorylation does not perturb the translational activation of these mRNAs. Collectively, all our observations support the overarching hypothesis that the initiation of translation in mouse oocytes primarily relies on CDK1-dependent phosphorylation of CPEB1, and not CPEB1 destabilization. This phosphorylation likely triggers polyadenylation that enhances the translation rate through ribosome recruitment. This CDK1-dependent phosphorylation is distinct from that induced by the AURKA/PLK1-dependent pathway. In a subsequent phase, both pathways induce hyper-phosphorylation and destabilization of CPEB1. Although our findings challenge the notion that CPEB1 destabilization is indispensable for the initial translational activation, it remains possible that destabilization and altered stoichiometry of CPEB1 play a role in the translational regulation of a specific subset of maternal mRNAs later during the MI-MII transition.

The role of the AURKA-mediated phosphorylation of CPEB1 has been controversial (Radford et al., 2008). Data in frog oocytes identified Ser 174 as the site phosphorylated by AURKA that is required for translational activation, whereas other sites are reported to be related to CDK1-mediated hyper-phosphorylation (Mendez et al., 2000a; Mendez et al., 2002). However, additional reports in frogs questioned this AURKA role (Frank-Vaillant et al., 2000; Keady et al., 2007). We have previously reported that MLN inhibition did not alter the CPEB1 phosphorylation state in mouse (Han et al., 2017); however, the oocyte culture conditions may have biased the results. In addition, conflicting data showed no effect or an effect of AURKA in pig oocytes (Komrskova et al., 2014; Nishimura et al., 2010). Available phosphoproteomics data did not detect phosphorylation of the residue that, in the mouse (Thr 171), corresponds to Ser174 in *Xenopus* CPEB1 (Cheng et al., 2022). Therefore, the activating phosphorylation in mouse oocytes likely corresponds to one of the mapped CDK1/MAPK sites, whereas the phosphorylations linked to destabilization are overlapping between frog and mouse (e.g. Ser207 mouse and Ser210 *Xenopus*).

In a previous report, we also found that CPEB1 phosphorylation was altered and the protein was stabilized (Aboelenain and Schindler, 2021). However, when an *Aurkc* 3′ UTR luciferase reporter was tested, translation was not observed. This result led to the conclusion that AURKA controls CPEB1-dependent translation. This conclusion conflicts with the findings described here. It is possible that the AURKA pathway controls only a subset of mRNAs for translation, perhaps dictated by subcellular localization or other UTR regulatory sequence elements. AURKA could also have different functions or interactions with CPEB1 during later meiotic transitions. Because *Aurka* cKO is associated with major dysfunction of the spindle and microtubule dynamics (Blengini et al., 2021), it is possible that localized translations in a compartment around the spindle may be disrupted in this genetic model (Waldron and Yajima, 2020; Pascual et al., 2020).

Consistent with the hypothesis proposed in previous reports, we conclude that hyper-phosphorylation of CPEB1 is the signal that triggers destabilization. We reached this conclusion because the time course of CPEB1 hyper-phosphorylation appearance precedes the timing of CPEB1 turnover. In addition, any condition that delays or prevents hyper-phosphorylation also prevents destabilization. The only observation inconsistent with this hypothesis is that MAPK inhibition causes CPEB1 stabilization while in the hyper-phosphorylated state. One explanation that reconciles this discrepancy is the possibility that MAPK inhibition interferes with mechanisms required for proteasome activation and ubiquitylation. Further experiments are required to test this possibility. Moreover, post-translational destabilization of CPEB1 may not be the only mechanism leading to a decrease in CPEB1 protein. We have shown that ribosome loading onto *Cpeb1* mRNA decreases with a time course not dissimilar to that shown for the CPEB1 decline (Luong et al., 2020) (Fig. S8). Therefore, it is most likely that the observed decline in CPEB1 protein is the combined result of decreased translation and destabilization of the previously synthesized protein.

Numerous reports have linked destabilization of CPEB1 with activation of translation. Although this may be true for frog oocytes (Mendez et al., 2002), all our data are inconsistent with this hypothesis in mouse oocytes. First, both genetic and pharmacological inactivation of the AURKA/PLK1 pathway leads to stabilization of CPEB1. Yet the increased CPEB1 levels are not associated with decreased translation of *Ccnb1* and *Mos* mRNAs. Second, stabilization of CPEB1 using a proteasome inhibitor stabilizes CPEB1 but has no effect on translational activation of *Ccnb1*. Last, and more importantly, mRNA translational activation precedes destabilization of CPEB1 by 3-4 h. This temporal dissociation strongly implies that the initial ribosome loading, reporter accumulation and endogenous protein accumulation are due to a crucial CDK1-dependent phosphorylation of CPEB1 and not to degradation at least up to or around metaphase I. Nevertheless, it remains possible that the AURKA-dependent destabilization of CPEB1 plays a role at the MI-MII transition. We could not directly investigate the effect of CPEB1 stabilization on the MI-MII transition because the *Cpeb1^fl/fl^* Zp3-cre and *Aurka* cKO, as well as the pharmacological inhibitors, all prevent anaphase I onset and polar body extrusion. In *Xenopus* oocytes, overexpression of a mutant *Cpeb1* resistant to degradation prevents the MI-MII transition and CCNB1 synthesis at this stage (Mendez et al., 2002). Conversely, overexpression of a stable CPEB1 only partially prevents the MI-MII transition in mouse oocytes (Sha et al., 2017).

Our genetic data show that the activation of translation of both *CcnB1* and *Mos* mRNAs is dependent on CPEB1. However, detailed analyses of the time courses of translational activation document significant temporal differences, with *Mos* translation preceding that of *Ccnb1*. Inspection of these two mRNAs shows that the cis-acting elements configuration in the 3′ UTR is similar but not identical to that described in *Xenopus* (Stebbins-Boaz et al., 1996; Mendez et al., 2002). The mouse *Mos* 3′ UTR includes a putative *Pum1* (TGTAGATAA), a *Zar1* (TTTGTGT) and two long polyU stretches identified as an embryonic CPE. Conversely, at least four functional CPEs have been mapped in the 3′ UTR of *Ccnb1*. Therefore, the different time course in mouse oocytes may be due to the presence, properties and location of CPEs, as proposed for *Xenopus* oocytes. Nevertheless, the presence of other regulatory elements may contribute to the difference in the timing of translation. In addition to a distinct initial timing of activation, translation between MI and MII follows a different pattern for the two mRNAs, with ribosome loading and the rate of translation of *Mos* reaching a plateau around 5-6 h or MI, whereas ribosome loading and translation of the *CcnB1* mRNA continues to increase between MI and MII (Fig. S2). This difference is reminiscent of the early and late translations described in *Xenopus* oocytes.

In summary, our data support the hypothesis that both the CDK1/MAPK and AURKA/PLK1 pathways are involved in CPEB1 regulation as they converge on the phosphorylation of this protein. In regulating the phosphorylation of this RBP, the two pathways have a role in translational regulation during meiotic maturation. Initial CPEB1-mediated translational activations are distal to CDK1 activation but are independent of AURKA/PLK1-mediated phosphorylations. Later during MI, both kinase cascades contribute to the destabilization of CPEB1, which is not required for translational activation. However, this late destabilization may contribute to further translational activation of *Ccnb1* mRNA during the transition from MI to MII. It therefore possible that a subset of translational activations at these later stages of meiotic maturation requires the activity of both kinases.

## MATERIALS AND METHODS

### Mice

All procedures involving mice were approved by the University of California, San Francisco's Institutional Animal Care and Use Committee (AN197697-00B). Animal care and use followed relevant guidelines and regulations. Mice were housed in a 12-12 h light-dark cycle with access to food and water *ad libitum*, under constant temperature. All animals used were of the *C57BL/6J* inbred strain. CPEB1-targeted mice were a gift from Dr Raul Mendez (Calderone et al., 2016) and were bred in our laboratory. Aurora kinase A (AURKA) conditional knockout mice have been described previously (Blengini et al., 2021) and were bred in the Schindler laboratory. Oocytes were shipped from the Schindler lab for western blot processing and mice were shipped for live reporter assay imaging. These mice were approved by the Rutgers University Institutional Animal Care and Use Committee (protocol 201702497).

### Oocyte collection and culture

Female mice (3-4 weeks old) were intraperitoneally injected with 5 IU pregnant mare serum gonadotropin (PMSG; MyBioSource, MBS173236) to induce follicle growth. After 44 h, the ovaries were dissected and cumulus-oocyte complexes (COCs) were collected in media containing HEPES-modified Eagle's minimum essential medium (Sigma-Aldrich, M2645) supplemented with 6 mM sodium bicarbonate (JT-Baker, 3506-1), 0.2 mM sodium pyruvate (Gibco, 11360070), 75 µg/ml penicillin+10 µm/ml streptomycin [Genesee Scientific Corporation (GenClone), 25-512], 3 mg/ml BSA (Sigma-Aldrich, SIAL-A3311) and a PDE inhibitor (1 µM cilostamide; Calbiochem, 231085). Using a glass pipette, aspiration was performed to remove the cumulus cells surrounding the eggs. Denuded oocytes were maintained at the GV stage in alpha-minimal essential medium (α-MEM) (Gibco) supplemented with 0.2 mM sodium pyruvate, penicillin-streptomycin and 1 µM cilostamide at 37°C under 5% CO_2_. After microinjection and overnight incubation, oocytes were transferred to α-MEM without cilostamide and allowed to mature. For GV state oocyte samples, oocytes were kept in cilostamide medium. For AURKA oocyte sample collection, oocytes were kept in MEM containing 2.5 µM milrinone (Sigma-Aldrich, M4659). Oocytes were then transferred to MEM without milrinone and allowed to mature. Oocytes were then collected at various time points. For AURKA/PLK1 pathway experiments, oocytes were preincubated for 1 h with 1 µM MLN8237 (Selleckchem, s1133) or 0.1 µM Bi2536 (VWR, 101757-124) before release from cilostamide. For 10 µM RO3306 (Selleckchem, s7747) and 20 µM UO126 (Selleckchem, s1102) experiments, the inhibitors were added after a 2 h incubation without cilostamide. The inhibitor experiments were carried out without oil cover.

### Construction of fluorescent protein reporters

The 3′UTR sequences of *Ccnb1* and *Mos* were obtained by amplification of mRNA from oocytes with appropriate primers (see Table S1) and fused with Ypet ORF. (C483S)-CDC25B coding sequences were also fused with Ypet ORF. The CDK1 FRET sensor was a gift from Jonathon Pines (Institute of Cancer Research, London, UK) (Addgene plasmid 26064). They were subcloned in the pCDNA 3.1 vector containing a T7 promoter and the constancy was confirmed via DNA sequencing. Linearized cDNAs were then transcribed *in vitro* to synthesize mRNAs with mMESSAGE mMACHINE T7 Transcription Kit (Ambion, AM1344) and purified using MEGAclear kit (Ambion, AM1908). The *mCherry* reporter was similarly produced and polyadenylated (150-200 nucleotides) using Poly(A) Tailing kit (Ambion, AM1350).

### Microinjection and time-lapse microscopy

Collected GV-arrested oocytes were injected with 5-10 pl of 12.5 ng/µl solution of the Ypet reporters along with a *mCherry* reporter or FRET reporter using a FemtoJet Express programmable microinjector with an Automated Leica microinjection Microscope System (Leica DMI 4000 B). After overnight pre-incubation in α-MEM with 1 µM cilostamide, oocytes were released from cilostamide and matured *in vitro* under time-lapse microscopy. Live cell imaging was performed under a Nikon Eclipse T2000-E equipped with a mobile stage and an environmental chamber at 37°C and 5% CO_2_. Filter set: dichroic mirror YFP/CFP/ mCherry 69008BS; Ypet channel (Ex: s500/20×49057 Em: D535/30 m 4728811); mCherry channel (Ex: s580/25×49829 Em: D632/60 m); and Cerulean channel (Ex: 430/25×49829 Em: 480/40 m 49287), for FRET channel (Ex: 430/25×49829 Em: D535/30 m 47281). Images were processed and fluorescence was quantified using MetaMorph (Molecular Devices). For reporter assay, YFP and mCherry channels were corrected by background. The ratio of Ypet fluorescence and the maximum level of mCherry signal were then plotted as an accumulation of translation activity. For the FRET assay, YFP, CFP and FRET channels were corrected by background. FRET channel was corrected for YFP bleed-through, and FRET was calculated as FRET channel/CFP channel.

### Western blot analysis

Oocytes at indicated times of incubation under maturing conditions were collected in 0.1% w/v PBS/PVP and mixed with Laemmli sample buffer supplemented with β-mercaptoethanol, proteinase inhibitor and phosphatase inhibitor. After boiling at 95°C for 5 min, the lysates were separated on 8% v/v or 12% v/v polyacrylamide gels and transferred to a polyvinylidene difluoride (PVDF) membrane (Millipore ISEQ00010). Membranes were incubated in 5% w/v milk buffer for 1 h at room temperature and incubated in primary antibody overnight at 4°C. Antibodies against the following proteins were used: CPEB1 (Abcam, ab73287, 1:1000), Cyclin B1 (Cell-Signaling, 4138, 1:1000), Aurora kinase (Cell-Signaling, 2914T, 1:500), MAPK (Cell-Signaling, 4377, 1:1000), α-tubulin (Sigma-Aldrich, T6074, 1:10,000), DDB1 (Abcam, ab109027, 1:4000) and T320-PP1 (Abcam, ab62334, 1:30,000). Membranes were washed in 1×TBST and incubated in the appropriate secondary antibodies (anti-rabbit, GE Healthcare, NA934V, 1:10,000; anti-mouse, GE Healthcare, NA931V, 1:5000) for 2 h at room temperature. Clarity Western ECL substrate (Bio-Rad, 170-5061) was used to develop the blots.

After multiple exposures, films were scanned into JPEG files and analyzed using ImageJ 1.53a. Images were inverted and tight square boxes were drawn around each band. If necessary, the boundaries of a band were defined using the ImageJ plot-profile function. The average background of the film was subtracted from the integrated intensity for each band and then the ratio of integrated intensity over intensity of α-tubulin or DDB1 was calculated. The values in Fig. 2C are the ratio of the loading-corrected intensity of the hyperphosphorylated species between experimental group and DMSO control. In Fig. 3B, the 7 h/4 h ratio of the loading corrected intensity of the phosphorylated/hyperphosphorylated band is reported for each lane.

### *In vitro* Cdk1 assay (PP1 assay)

Thirty oocytes were collected in 30 µl of 2× kinase buffer (100 mM HEPES, 30 mM MgCl_2_, 2 mM EGTA, 10 mM CaCl_2_, 2 mM DTT, 2 µg/ml leupeptin, 2 µg/ml aprotinin and 2 µM okadaic acid). The oocytes were then lysed by freeze-thawing them three times in liquid nitrogen. Subsequently, 0.1 mM ATP, 10 mM DTT and 2 µg of the recombinant peptide PP1-GST were added to the lysates as the substrate. The lysates were incubated at 30°C for 30 min, and the reactions were stopped by boiling them at 95°C for 5 min after adding Laemmli sample buffer (BioRad, 161-0747) supplemented with β-mercaptoethanol (Gibco, 21985023). Finally, CDK1 activity was assessed by measuring the phosphorylated T320 of the total substrate using western blot analysis.

### Statistical analysis

All data are shown as mean±s.e.m. All statistical analyses were carried out using the Graph Pad Prism 9 software. Unpaired Student's *t*-tests and Mann–Whitney tests were performed and a *P* or *Q* value of less than 0.05 was considered statistically significant.

## Supplementary Material

10.1242/develop.202712_sup1 Supplementary information
