# Supplementary material for: Multiple intersecting pathways are involved in CPEB1 phosphorylation and regulation of translation during mouse oocyte meiosis
Source: Development. 2024 Jun 4;151(11):dev202712. doi: 10.1242/dev.202712 (PMC11190569; doi:10.1242/dev.202712)
Supplement: Supplementary information [file develop-151-202712-s1.pdf]

Table S1. Primer sequences used to generate the 3' UTRs used in the reporter assay.

**Name**	**Prime (5'-3')**
Ccnb1 FW	CACCATCACCATTGACTCCAATAGAC
Ccnb1 Rev	GATCAGCGGGTTTAAACAAGCTTTCC
Mos FW	CAATAATTCTAGACTCCATCGAGCCGATGTAGAG
Mos Rev	CCACCTGGATCCGAAGTTCGTGGTAACTTTATTTC
mCherry FW	GAACGGCCACGAGTTCGAGA
mCherry Rev	CTTGGAGCCGTACATGAACTGAGG
FRET FW	TTGGAGCGCTTGACCTTGGGCTAAGGATCCACCGGATCTAGATAACTGATCATAAT
FRET Rev	TCGGCATGGACGAGCTGTACAAGGGCGGCGGCTTGCCACCATTGGAGCGC
Ypet Rev	GATCCGGTGGATCCTAAAGATCTCTTATAGAGCTCGTTC
T7 FW	GAGAACCCACTGCTTAC
Vector FW	CTTGACGAGTTCTTCTGAGCGGGAC
Vector Rev	GTCCCGCTCAGAAGAACTCGTCAAG

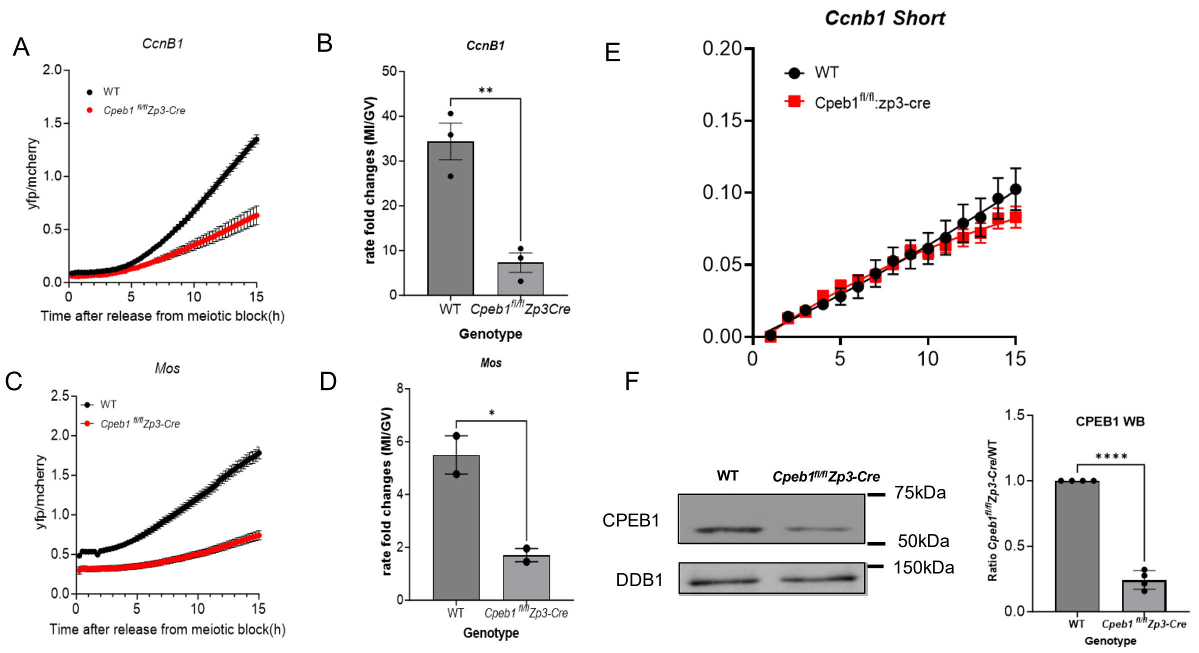

Fig. S1. CPEB1 is required for translational activation of *Mos* and *CcnB1* mRNAs.

(A-D) GV-arrested oocytes were injected with *CyclinB1 (Ccnb1)* or *Mos* reporter together with polyadenylated *mCherry* reporter. After overnight recovery with 1µM cilostamide (PDE inhibitor), oocytes were released from cilostamide and matured. YFP and mCherry signals were recorded by time-lapse microscopy every 15 min for 15 hrs. The YFP/ mCherry signal ratio for each oocyte was plotted and data are shown as the mean ± SEM. The bar graph shows the ratio of translation rate between GV and MI stages. Each bar is the mean ± SEM of three or more independent biological replicates. Two-tailed unpaired Student's test was used to evaluate statistical significance (*P<0.05, **P<0.01). (E) Translation of a short CPEB1 reporter lacking all CPE regulatory elements. Data are the mean ± SEM of 14 WT and 34 KO oocytes. (F) Residual CPEB1 protein expression in CPEB1$^{fl/fl}$Zp3-cre mice. Western blot was performed on lysates of 30 oocytes from wild-type (WT) and CPEB1$^{fl/fl}$Zp3-cre mice. A graph reports the quantification of the western blot from four independent biological replicates. The data are the mean ± SEM of the CPEB1/DDB1 ratios plotted as the changes over the control. Two-tailed unpaired Student's test was used to evaluate statistical significance (****P<0.0001).

Development • Supplementary information

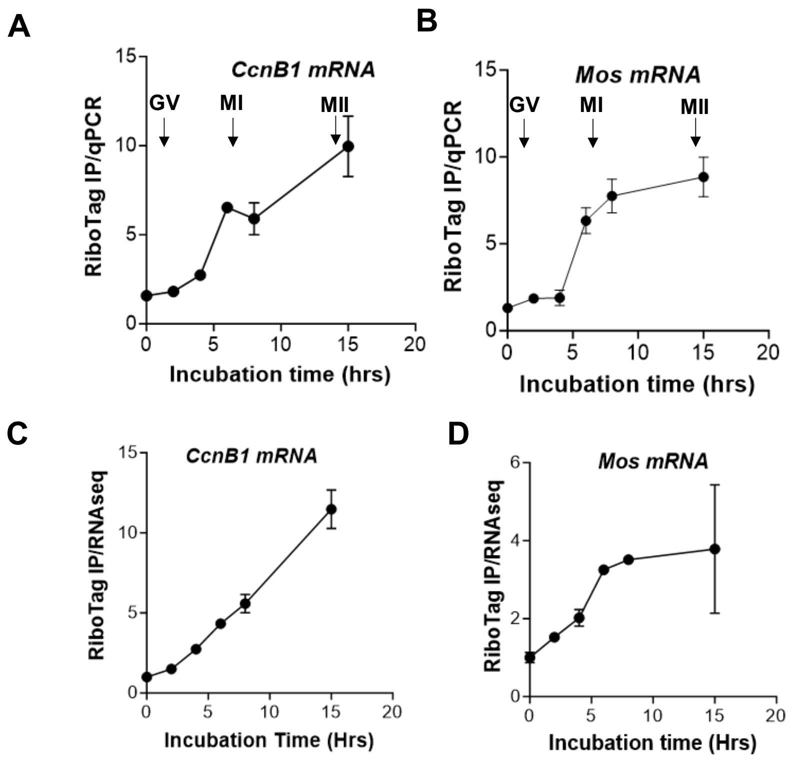

Fig. S2. Comparison of the time course of ribosome loading onto the endogenous CcnB1 and Mos mRNAs in mouse oocytes.

Ribosome loading was measured either by RiboTag IP/qPCR (A,B) or RiboTag IP RNAseq (C,D). Each point is the mean ± SEM of triplicate biological replicates for the RiboTag IP/qPCR. Duplicate determination and range is reported for the RiboTag IP/RNAseq. The data are taken from Luong et al., 2020 or Han et al., 2017.

Development • Supplementary information

A

Potential phosphorylation sites in mouse CPEB1

**Position In the sequence**	**Peptide sequence**	**Fold change MI/GV**	**Notes**
S4	MAF **S**LEEAAGRIKDCWDNQ	ot detected in	nknown
S43	S NIFRRINAILDD **S** S CTTPINRGI	ot detected in	otential 1 substrate
S207	SGS SS I**S** S GNGPRD	ot detected in	otential PK*
S245	S ALAAVAP **S**PTS PKRWPGAS WP	21.63363	otential PK
S85	ETVTSRMLFPTSAQE **S**PRGLPDANGLCLGLQ	6.883888	otential Cd 1
S181	S RPI SRSS **S** SDSDTSGFSSGS	0.498547	otential PK*
T52	NAI S CT **T**PINRGIHDQLPDFQD	ot detected in	ound amon PLK1 and Aur substrates

B

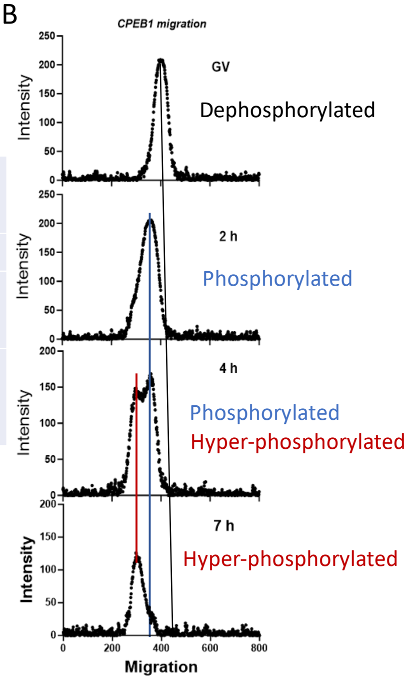

Fig. S3. Multiple CPEB1 phosphorylation sites and the generation of species with different SDS-PAGE in mouse oocytes

(A) The table summarizes the potential phosphorylation sites and the relative changes during the first meiotic division (fold change MI/GV) in mouse CPEB1 determined by a phosphoproteome approach (Ceng et al.,2022). Residues with the highest probability of being phosphorylated are reported in red. (B)The image shows the time-dependent shift in the CPEB1 immunoreactive band migration in western blot. Wild-type (WT) oocytes were allowed to mature *in vitro* for the indicated times. At the end of the incubation, oocytes were harvested, lysed, and fractionated on SDS-PAGE. Western blot with a CPEB1 antibody was performed on lysates of 30 oocytes. Image J quantification was done on lanes from the same gel. Lines of different color are drawn to mark the migration of the dephosphorylated (Black), Phosphorylated (Blue) and hyperphosphorylated (Red) forms.

Development • Supplementary information

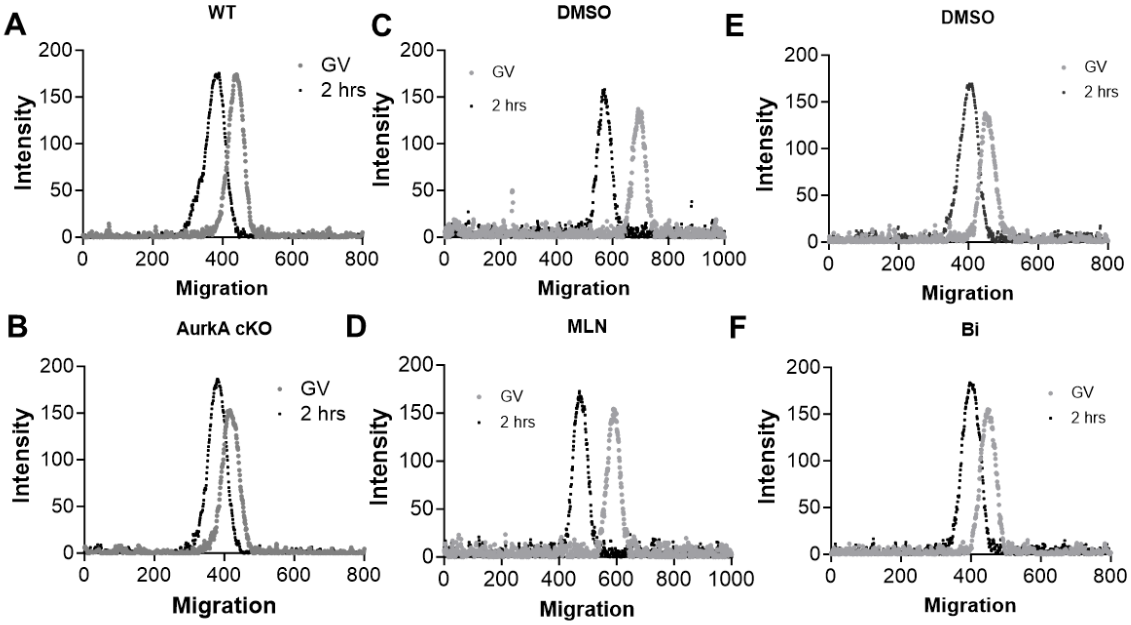

Fig. S4. Effect of AurkA genetic ablation or inhibition of AurkA and PLK1 kinases on the mobility shift of the CPEB1 protein.

WT oocytes and oocytes from the AurkA conditional KO were used in A and B, respectively. WT oocytes incubated with either DMSO control, MLN8237 (1 uM), or Bi 2536 (0.1 uM) were used for panel C,D and E,F, respectively. All oocytes were harvested while in GV or allowed to mature for 2 hrs. at the end of the incubation, oocytes were harvested, lysed, and lysates fractionated on SDS-PAGE. Western blot was performed with a CPEB1-specific antibody. The migration of the immunoreactive band was assessed using ImageJ Plot using the Profile function. In grey is the migration of the band from oocytes in GV (dephosphorylated form) and in black is the migration after 2 hrs of maturation. A shift in mobility corresponding to the phosphorylated forms is present in all the conditions tested.

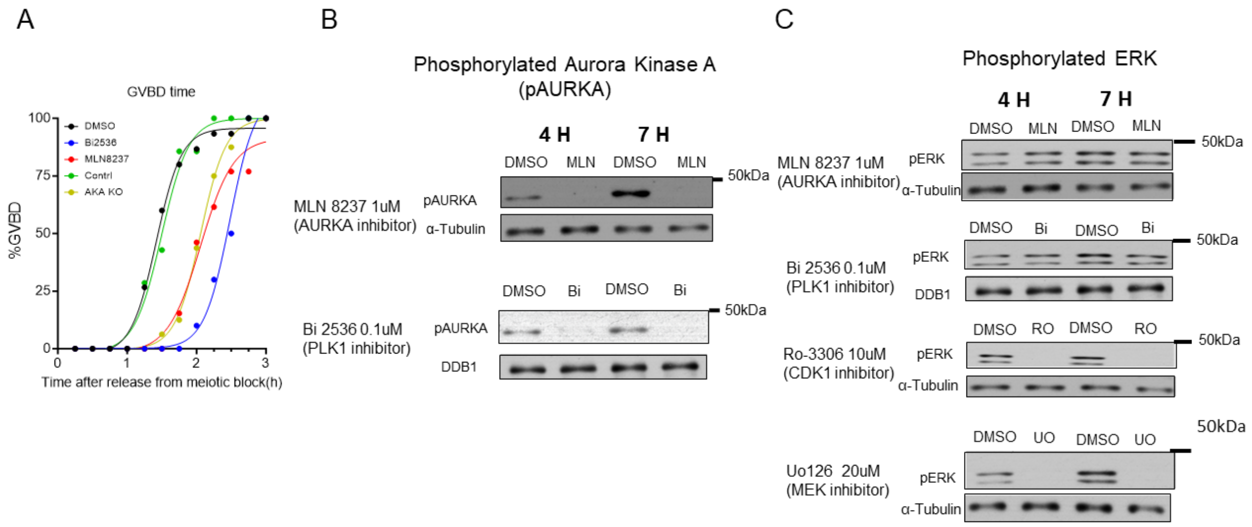

Fig. S5. Delay in GVBD time and the control for the efficacy of the different treatments

Time-lapse analysis of the timing of GVBD in untreated oocytes or oocytes exposed to DMSO, ML8237, or Bi2536. The time course of GVBD in oocytes from the AurkA cKO is also included (B) Representative western blot image showing the efficacy of MLN8237 and Bi2536 treatment in blocking the phosphorylation of Aurora Kinase A (AURKA). (C) Representative western blot images showing the effect of different treatments on the phosphorylation of ERK1/2. Alpha-Tubulin or DDB1 was used as a loading control. Western blot analysis was conducted on lysates of 30 oocytes. Several blots from Fig. 3 were stripped and reprobed with the antibodies shown here.

Development • Supplementary information

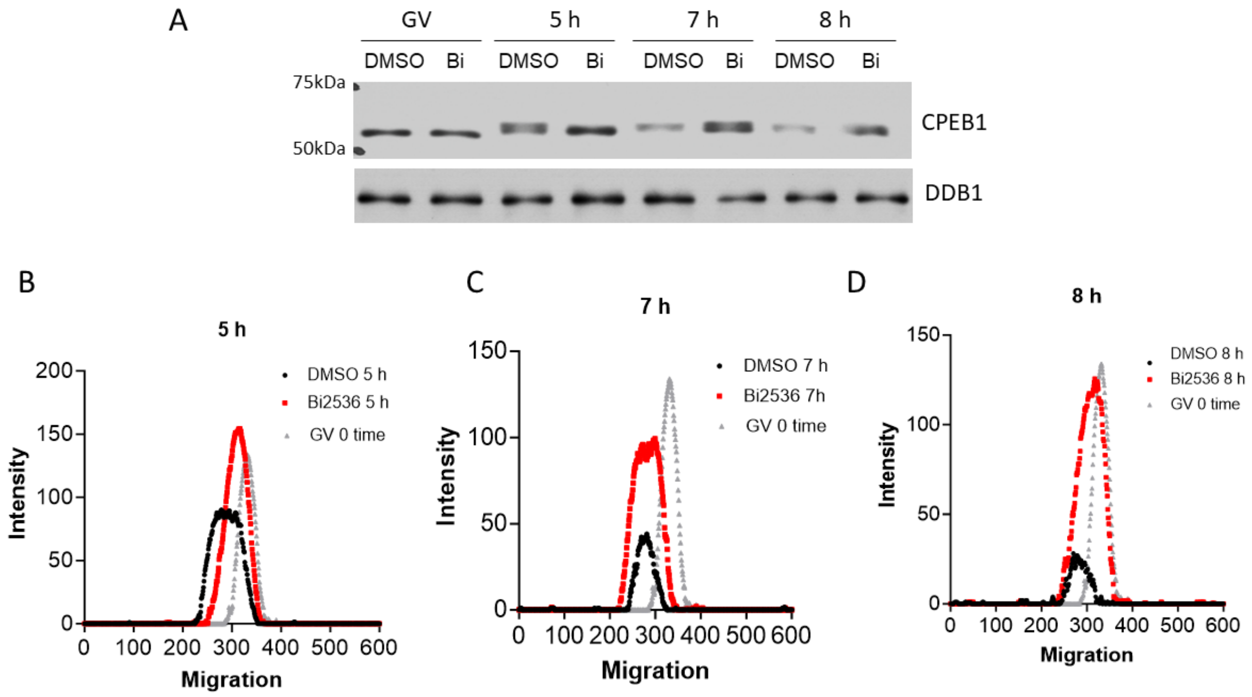

Fig. S6. Inhibition of PLK1 stabilizes the phosphorylated CPEB1 species.

(A) A representative western blot of the time course of CPEB1 phosphorylation in oocytes treated with DMSO or Bi2536 (30 oocytes/ lane). DDB1 was used as a loading control. (B) Graphs of the migration of the Cpeb1 immunoreactive band during oocyte maturation in oocytes treated with either DMSO (black symbol) or Bi2536 (Red symbol). The grey symbols represent the migration of the CPEB1 immunoreactive band in oocytes in GV, providing a control for the dephosphorylated protein. The Bi2536 treatment prevents or delays the phosphorylation of CPEB1 and causes its stabilization at 7 and 8 hrs of oocyte maturation.

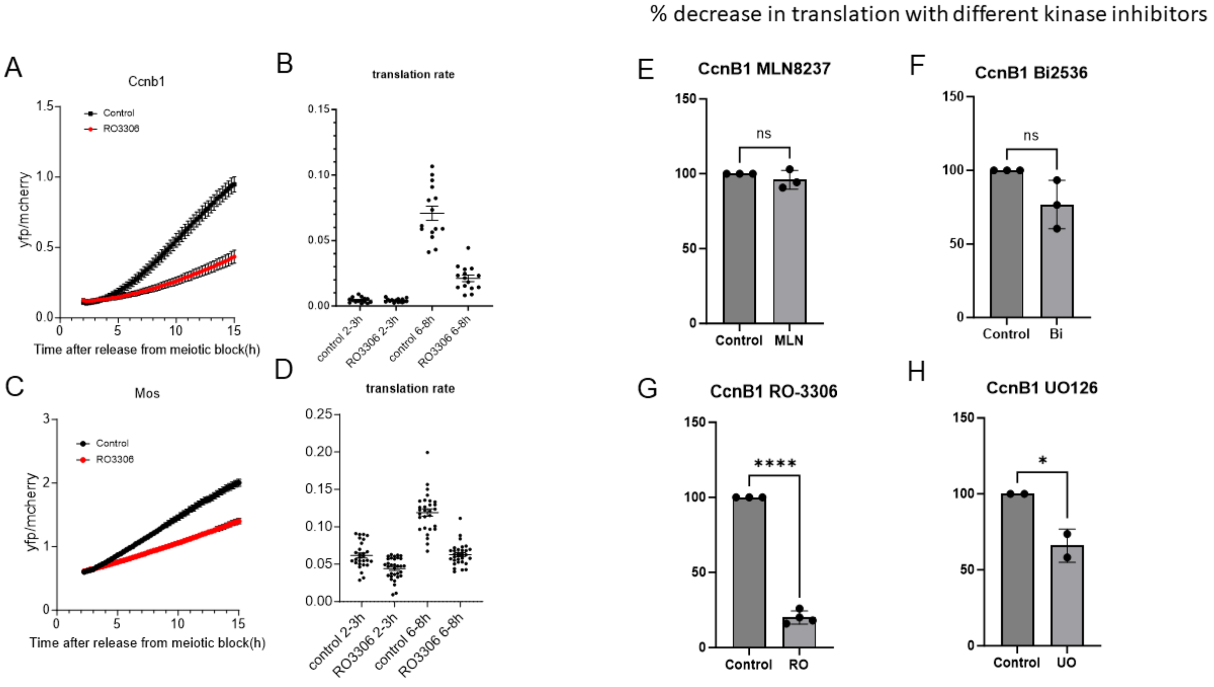

Fig. S7. CDK1 inhibition causes decrease in translation of *Ccnb1* and *Mos*

(A-D) GV-arrested oocytes were injected with *CyclinB1 (Ccnb1)* or *Mos* reporter and polyadenylated *mCherry*. After overnight incubation with 1µM cilostamide (PDE inhibitor), oocytes were released from cilostamide and matured. YFP and mCherry signals were recorded by time-lapse microscopy every 15 min for 15 hrs. The YFP/ mCherry signal ratio for each oocyte was plotted. The translation rate for each oocyte was calculated by linear regression of the reporter data within the indicated window. All data are shown as the mean ± SEM. (E-H) The graphs show the % decrease in translation of *Ccnb1* with different kinase inhibitors compared to the DMSO control. The data are plotted with the mean ± SEM and each point represents a different experiment and biological replicate. Two-tailed unpaired Student's test was used to evaluate the statistical significance (ns; not significant, *P<0.05, ****P<0.0001).

Translation of Cpeb1 and CPEB1 protein levels decrease at comparable times

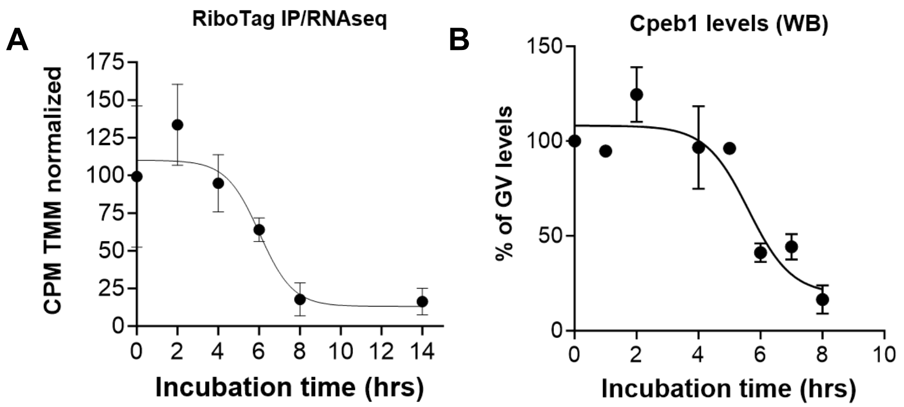

Fig. S8. The translation of the *Cpeb1* mRNA and the CPEB1 protein levels decrease with comparable time courses.

(A)The time course of ribosome loading onto the endogenous *Cpeb1* mRNA in mouse oocytes was measured by RiboTag IP/RNAseq. Each point is the mean + SEM of 3 to 5 biological replicates. (B) Western Blot Analysis of the levels of Cpeb1 during oocyte maturation. Each point is the mean + SEM of 2-3 different biological replicates. The points without brackets are a single measurement.

Development • Supplementary information

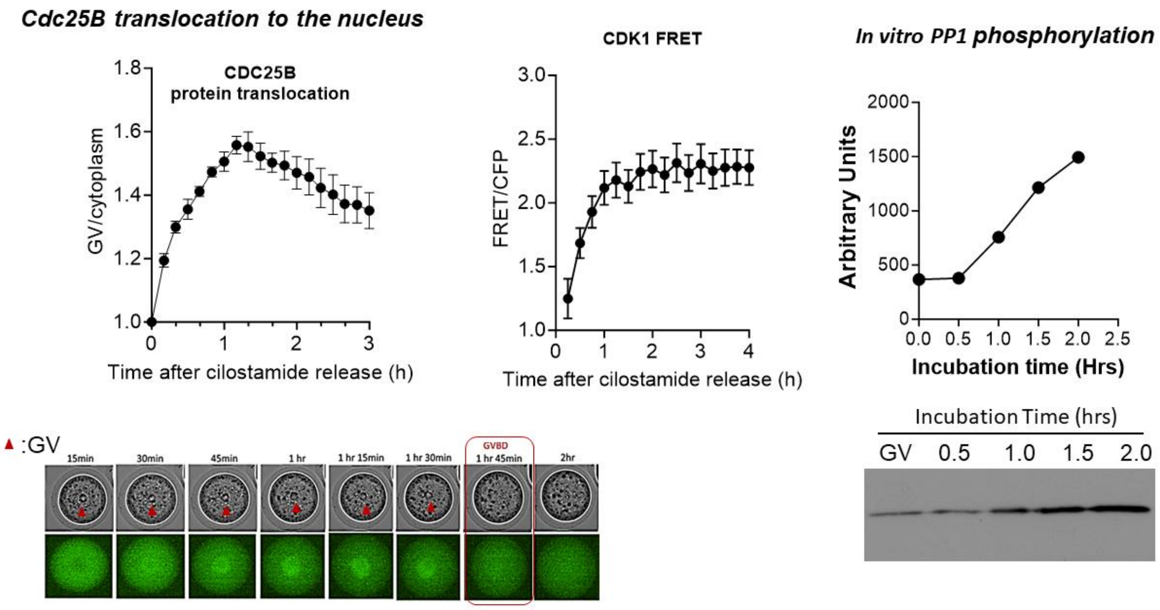

Fig. S9. Examples of measurements of the translocation of Cdc25B into the nucleus, of endogenous Cdk1 activity measured by FRET, and CDK1 activity measured in oocyte extracts at different times of maturation.

Development • Supplementary information
